# Response Patterns and Behavior of Seropositive Blood Donors: A Comprehensive Analysis

**DOI:** 10.7759/cureus.67462

**Published:** 2024-08-22

**Authors:** Arthi R, Soundharya V, Suresh Kumar I, Hari Haran A, Sahayaraj James

**Affiliations:** 1 Transfusion Medicine, Saveetha Medical College and Hospital, Saveetha Institute of Medical and Technical Sciences, Chennai, IND

**Keywords:** donor counselling, donor recall, safe transfusion practices, blood donation, transfusion transmitted infections

## Abstract

Background

Blood transfusion services are vital in healthcare, ensuring a steady and safe supply of blood for patients in need. Identifying seropositive blood donors and understanding their response patterns and behaviors are critical for improving the safety and efficacy of blood transfusion practices.

Aim

Our study aims to determine the response rate and pattern of blood donors who test reactive for transfusion-transmitted infections (TTIs) and to understand the attitudes of reactive blood donors toward post-donation notification, follow-up, and counseling.

Materials and methods

Our study is retrospective audit analyzing the data record for a period of 24 months from October 2021 to October 2023 in the transfusion medicine department of a tertiary care hospital in southern India. All donations were screened for hepatitis B, hepatitis C, HIV, malaria, and syphilis.

Results

Of the total 8,276 donations during the study period, 117 (1.41%) were reactive for various TTIs. The highest prevalence was hepatitis B (0.61%), followed by hepatitis C (0.38%), then HIV (0.22%) and syphilis (0.14%). Of all the TTI reactive donors, 82 donors (70%) responded after communication, and the remaining 30% could not be contacted by any mode of communication. The most common reason for non-communication was the fabricated postal address given by the donors. Of the donors approached, 57 (48.7%) reinstated to our blood bank for further counseling and specific treatment. The donor’s busy schedule and out-of-city residence were the main reasons for non-compliance with the follow-up protocol.

Conclusion

Informing donors of their serological status and providing post-donation counseling are crucial elements of blood transfusion protocol. Necessary knowledge about TTIs are to be instilled to the donors during the blood collection procedure. Moreover, its crucial for the donor to provide accurate demographic details aiding follow up and easy access during times of managing blood inventory.

## Introduction

Blood transfusion services play a crucial role in healthcare, ensuring a stable and safe supply of blood for patients in need. One key aspect of blood donation is the identification of seropositive donors. Understanding the response patterns and behavior of seropositive donors is essential for enhancing the safety and efficacy of blood transfusion practices. Response pattern refers to how donors who test positive react to their results and engage in counseling to understand the policies around blood donation. The behavior of seropositive donors is influenced by their perception of risk and the stigma associated with their status. Some donors may conceal their health history due to fear of social stigma, which can further complicate their willingness to seek help or change their behaviors.

Multiple-transfused patients face a higher transfusion-transmitted infection (TTI) risk, as noted in Mittal et al.'s study, which showed 12.5% infection [1]. Despite mandatory screening in India for hepatitis B virus (HBV) since 1971, HIV since 1989, hepatitis C virus (HCV) since 2001, malaria, and syphilis, TTIs persist due to infections during the window period [2]. To enhance prevention, informing and counseling donors about their TTI-positive results can deter infective donors from donating in the future.

The Indian Action Plan for Blood Safety involves counseling donors about TTIs before donation and providing the option to learn their infective status with consent [3]. Donors with reactive screening results are notified through various means like letters and telephonic calls and encouraged to undergo counseling and repeat testing. Despite technological advancements like the p24 antigen and nucleic acid amplification test, there is a challenge of increased false-positive cases, emphasizing the need to balance sensitivity with avoiding unnecessary anxiety in notified donors.

There is a lacuna of information regarding donor counseling and referral follow-up in India [4]. Blood banks often discard TTI-reactive blood without notifying donors due to resource and counseling constraints [5]. Unfortunately, many notified donors either do not respond or fail to follow up, leading to some reactive donors to persist in donating blood [6].

Detecting reactive donors and confirming positive cases is essential for preventing the transmission of TTIs. This process allows blood banks to temporarily or permanently defer individuals who pose a risk of transmitting infections through blood donation. Furthermore, it enables the re-entry of donors who were incorrectly identified as reactive during their initial screening, ensuring fair treatment and maintaining a safe blood supply. This study aims to understand the attitudes of reactive blood donors toward post-donation notification and counseling. The goal is to empower blood banks to enhance their role in ensuring safe blood for patients by deterring reactive donors. The findings may also contribute to promoting the significance of self-deferral.

## Materials and methods

This is a retrospective study done in the Department of Transfusion Medicine, Saveetha Medical College and Hospital, Chennai. All data were obtained from records maintained in the department for a period of 24 months from October 2021 to October 2023. Prior to donation, all donors were screened as per the department’s standard operating procedures (SOPs) and criteria for selection of donors as per the Drugs and Cosmetics Act of 1945 [2]. The phone number and complete postal address of each donor were noted at the time of donation. Consent to inform about any abnormal result was obtained.

All donations were screened for HIV, HBV, HCV, malaria, and syphilis. HIV, HBV, and HCV were tested using the VITROS ECi/ECiQ Immunodiagnostic System (Ortho Clinical Diagnostics, Raritan, NJ) by enhanced chemiluminescence immunoassay (CLIA) technique. HIV was screened by the detection of antibodies to HIV type 1, including groups M and O, and HIV type 2 (anti-HIV-1 and anti-HIV-2) in donor serum. Hepatitis B was screened by the detection of hepatitis B surface antigen (HBsAg) in donor serum. Hepatitis C was screened by the detection of antibodies to HCV (anti-HCV) in the patient serum. All the donations that showed optical density (OD) values above the cut-off, as calculated by the manufacturer’s instructions, were considered reactive. According to the manufacturer's kit insert, the VITROS test has a sensitivity and specificity of 99.7% and 99.9% for detecting HIV antibodies, 97.5% and 99.7% for detecting HBsAg, and 99.5% and 98.2% for detecting anti-HCV, respectively.

Syphilis was tested by rapid plasma reagin (RPR) card (carbogen) to detect anti-lipoidal antibodies. Carbogen has a diagnostic specificity of 98%, sensitivity of 86% for primary syphilis, and 100% for secondary syphilis. Malaria was tested using a rapid card test (parabank) to detect the presence of Pan malaria genus-specific parasite lactate dehydrogenase (pLDH) released from the parasitized blood cells. Parabank has a sensitivity and specificity of more than 90% for all species of malaria.

Reactive donors were called to the blood bank by telephone call and letters to their postal addresses. As per institutional SOP, the donor is notified of any anomalous results three times before their noncompliance is classified as non-responders. Confidentiality is maintained by informing the donor solely about the detection of an abnormal result and advising them to contact the blood bank. When they reported to the blood bank, they were informed about their abnormal test results and the need for further testing. They were also given one-to-one counseling. As per institutional protocol, hepatitis B, hepatitis C, and malaria reactive donors were referred to the medicine department, syphilis reactive to the dermatology and venereal diseases department, and HIV reactive to the Integrated Counseling and Testing Center (ICTC) of the hospital. Further testing and confirmation are done at the respective departments.

Inclusion criteria

Our study included all whole blood donations that were screened for TTIs during the study period.

Exclusion criteria

Incomplete collections, where the quantity was less than 10% of the standard blood bag volume, making them not suitable for use, were not included in the study as these bags were not screened for TTIs. Donors who were screened for single-donor plasma (SDP) were not included, as they were informed of their TTI screening results before donation.

Data collected was entered in Microsoft Excel (Microsoft® Corp., Redmond, WA) and analyzed based on donors who could be communicated, reasons for non-communication, donors who responded to communication, and reasons for not responding. Donor details were kept confidential. Local management’s clearance was taken before data compilation. Only data related to blood donation and screening were retrieved from the registers and analyzed, and institutional ethical committee clearance with registration number 067/10/2023/PG/SRB/SMCH was obtained for this study.

## Results

There was a total of 8,276 donations over the study period of two years. On screening for the five TTIs, 117 (1.41%) donations were found to be reactive. The seropositivity rate is the number of donors who tested positive for the TTI among all the donors screened. The highest prevalence was HBsAg (0.62%), followed by HCV (0.39%), then HIV (0.23%), and syphilis (0.14%), as shown in Table 1. There were no cases of malaria reported. There were three cases (0.03%) of coinfection with HBV and HCV.

**Table 1:** Seropositivity rate of TTIs HIV - detection of antibodies to HIV type 1, including groups M and O, and HIV type 2 (anti-HIV-1 and anti-HIV-2). Hepatitis B - detection of HBsAg. Hepatitis C - detection of antibodies to hepatitis C virus (anti-HCV). Syphilis - RPR test to detect anti-lipoidal antibodies. Malaria - rapid card test to detect the presence of pLDH. HBsAg, hepatitis B surface antigen; HCV, hepatitis C virus; pLDH, parasite lactate dehydrogenase; RPR, rapid plasma reagin; TTI, transfusion-transmitted infection

S. no.	TTI	No. of seropositive donors	Seropositivity rate (95% confidence interval)
1	HIV	19	0.23% (0.15 to 0.36)
2	HBsAg	51	0.62% (0.47 to 0.81)
3	HCV	32	0.39% (0.27 to 0.55)
4	Syphilis	12	0.14% (0.08 to 0.25)
5	Malaria	0	0%
6	HBsAg + HCV	3	0.03% (0.01 to 0.11)

The age group of 26 to 35 years was the most commonly affected (44 donors). There was also a considerable number of 37 cases in the age group of 36-45 years, as shown in Table 2. The reduction of reactive donors at the extremes of the age groups is probably due to the lower number of donors in these age groups.

**Table 2:** Age distribution of TTI reactive donors HBsAg, hepatitis B surface antigen; HCV, hepatitis C virus

Age	HIV	HBsAg	HCV	Syphilis	HBsAg + HCV	Total
18-25	1	6	2	2	0	11 (9.4%)
26-35	8	15	14	5	2	44(37.6%)
36-45	5	18	10	3	1	37 (31.6%)
46-55	4	9	6	1	0	20 (17%)
56-60	1	3	0	1	0	5 (4.2%)

**Figure 1:**
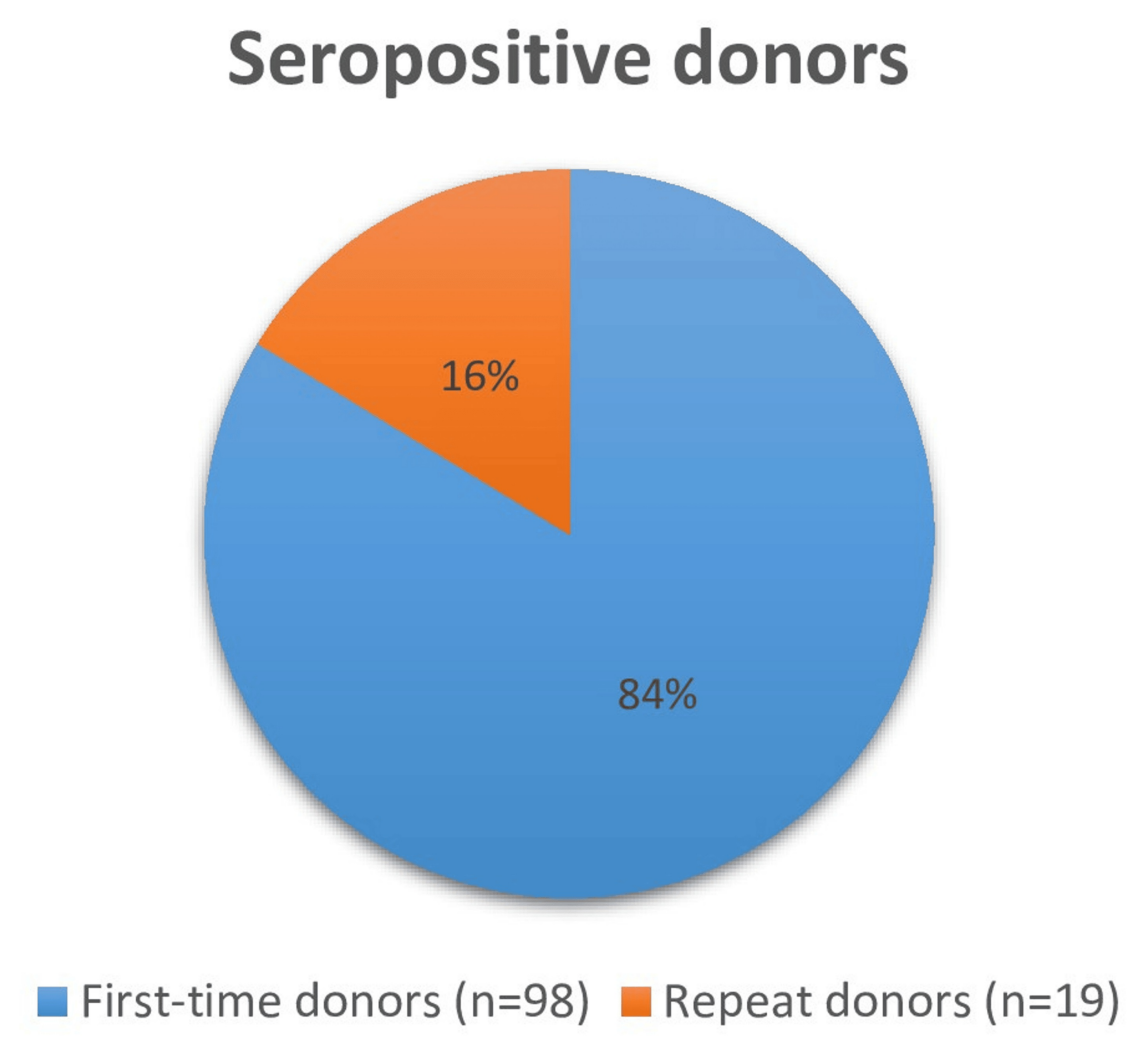
Reactive donors according to the number of donations

Seropositivity rate was higher in first-time donors (98 donors - 83.7%), and all responding donors were first-time donors, as seen in Figure 1.

**Figure 2:**
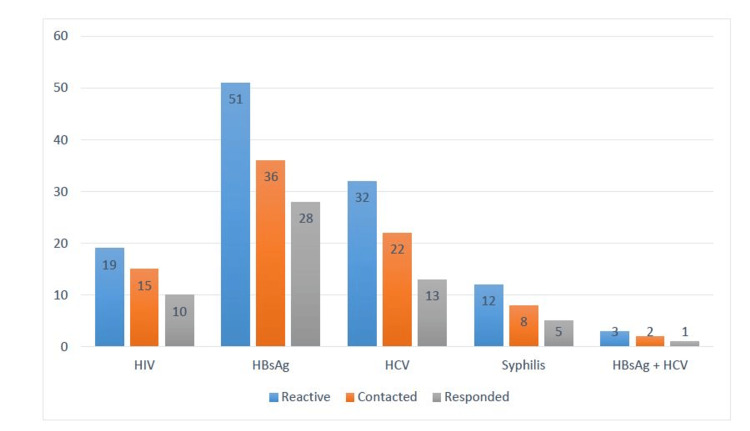
Contact and response rate among transfusion-transmitted infection reactive donors

Serozreactive donors are classified as donors who could be communicated and donors who could not be communicated. Of all the TTI reactive donors, 82 donors (70%) could be contacted by telephone calls and letters, and the remaining 35 donors (30%) could not be contacted by any mode of communication. The contact and response rate among TTI reactive donors is shown in Figure 2.

**Figure 3:**
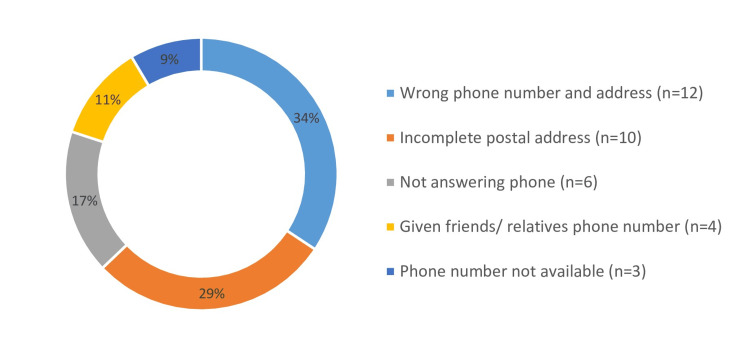
Reasons for non-communication

Most common reason of non-communication was wrong phone numbers and address given by the donors. The other reasons for non-communication are shown in Figure 3.

**Figure 4:**
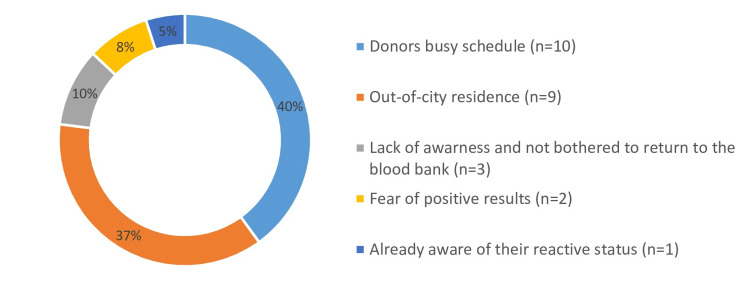
Reasons for non-responding seroreactive donors

Donors who were communicated were again divided into donors who responded/returned back as responders and donors who did not respond or return back as non-responders, as shown in Table 3. The donors’ busy schedules and out-of-city residences were the main reasons for not returning back for counseling and further management. The other reasons for non-responding seroreactive donors is shown in Figure 4.

**Table 3:** Responders among TTIs reactive donors HBsAg, hepatitis B surface antigen; HCV, hepatitis C virus; TTI, transfusion-transmitted infection

TTIs	Male		Female		Total	
	No. of donors	Responders	No. of donors	Responders	No. of donors	Responders
HIV	19	10	0	0	19	10 (52.6%)
HBsAg	50	27	1	1	51	28 (54.9%)
HCV	32	13	0	0	32	13 (40.6%)
Syphilis	12	5	0	0	12	5 (41.6%)
Malaria	0	0	0	0	0	0
HBsAg + HCV	3	1	0	0	3	1 (33.3%)
Total	116	56	1	0	117	57 (48.7%)

## Discussion

Ensuring safe blood transfusion necessitates not only comprehensive pre-donation counseling and TTI screening but also includes post-donation counseling and notification of donors identified as TTI reactive. Informing a blood donor about abnormal test results constitutes a highly sensitive and crucial aspect of post-donation counseling, given its potential psychological and social impacts [7]. The fundamental principles governing donor notification should encompass the timely, accurate, and confidential provision of information in a manner that minimizes anxiety and fosters comprehension [8].

The response rate in our study was 48.7%, which was lower than similar studies conducted in India, like Roshan et al. and Patel et al., who had a response rate of 63.5% and 60.36%, respectively [9,10]. However, studies by Harjot et al. had a lower response rate of 27.45% [11]. The comparison of response rates in various studies has been shown in Table 4. In studies conducted by Tynell [12] in Sweden, the response rate was reported at 88%. In these studies, prospective blood donors were equipped with essential information and underwent screening for infectious agents before the actual donation process took place. Considering the low response rate in our country, it becomes crucial to reevaluate the pre-donation donor screening policy. Utilizing sensitive, rapid tests, which can be completed in just half an hour, allows for the immediate identification of reactive donors. This swift identification enables prompt initiation of subsequent treatment, offering a protective measure for society.

**Table 4:** Comparison of response rate among seropositive donors in various studies HBsAg, hepatitis B surface antigen; HCV, hepatitis C virus; TTI, transfusion-transmitted infection

TTIs	Harjot et al. [11]	Roshan et al. [9]	Patel et al. [10]	Present study
HIV	18.18%	54%	52.54%	52.6%
HBsAg	32.75%	58.9%	19%	54.9%
HCV	25.31%	70.7%	20%	40.6%
Malaria	00	00	00	00
Syphilis	26.7%	32.9%	15%	41.6%
Average	27.45%	63.5%	60.36%	48.7%

Wrong or incomplete phone numbers and addresses were the main reasons for not being able to communicate with the donors. Ensuring donor forms are completely filled becomes crucial to mitigate this aspect. Implementing digital forms that require all fields to be filled out before submission can help resolve this problem. Communicating the results with the donor is crucial for further follow-up and treatment.

Sharma et al. [13] identified a significant lack of awareness among donors regarding the concept of the window period. Many donors expressed the belief that it was acceptable to donate blood, even after engaging in high-risk behavior, under the assumption that the donated blood would undergo thorough testing for infectious agents and, if tested positive, it would be appropriately discarded.

Roshan et al. explored the phenomenon of individuals seeking tests by utilizing blood donation as a means for accessing free testing. [9] The study revealed that these potential test-seekers were cognizant that a notification from the blood bank clinic likely indicated a reactive screening test. In the context of developing countries like India, an additional category of donors, known as professional donors, who donate blood in exchange for monetary benefits, also warrants consideration. These donors are well-informed about the advantages of the testing process-being, both cost-free and confidential. Importantly, they understand that participating in the testing will not impact their daily lives due to the strict cultural taboos prevailing in society.

The primary reasons donors did not return to the blood bank for counseling were their busy schedules and out-of-city residence. To address this, donors should be followed up at a more convenient time. Additionally, arrangements could be made for them to attend counseling sessions in their own city. Donors who actively participate in counseling derive several benefits compared to those who do not engage after receiving notification [14]. Counseling sessions serve as a platform where donors are not only encouraged to inquire about their health status but also have the opportunity to dispel any myths and alleviate anxieties they may have. Moreover, during these sessions, donors receive detailed information about their responsibilities toward society and their partners and are educated about the various available treatment options for the specific disease in question. In contrast, donors who neglect counseling pose a continued threat to public health, their families, and the integrity of blood transfusion services.

Public awareness programs promoting blood donation should be integrated with initiatives to raise awareness about TTIs and the accessibility of their treatment. Utilizing banners, signboards, and conferences can effectively attract individuals from high-risk communities to hospitals for necessary treatment. Moreover, educational materials, Information, Education, and Communication (IEC) materials, as well as posters addressing high-risk activities and TTIs, should be strategically placed in donor screening rooms. This approach aims to encourage self-deferral among potential donors belonging to high-risk groups, promoting a proactive and responsible contribution to blood donation.

100% follow-up of reactive blood donors offers crucial benefits, including improved diagnosis and treatment by ensuring confirmatory testing, timely referrals, and prevention of future infection transmission. It enhances donor education and counseling by informing them about their test results, assessing risk factors, and addressing misconceptions. This approach also reduces donor loss by distinguishing between true and false positives, allowing eligible donors to return, and retaining valuable donors. Additionally, it improves blood safety by identifying and removing potentially infectious donations, informing blood screening strategies, and enhancing overall safety. In essence, comprehensive follow-up safeguards donor health, ensures blood safety, and minimizes unnecessary deferral.

The limitations of the study include its retrospective design, which prevents it from identifying changes in the behavior of reactive donors over time and limits the ability to observe temporal dynamics. Cultural factors can significantly influence behavior and may not be accounted for in the study. The limited sample size is not representative of the broader population of seropositive individuals, which restricts the applicability of the study's findings to the general population.

## Conclusions

To attain a 100% response rate from contacted reactive donors, it is imperative to educate donors during the donation process about the different TTI and screening tests conducted. Equally significant is conveying the importance of promptly informing them of the test results. Moreover, it holds equal weight to ensure that donors comprehend the critical role of providing accurate and comprehensive demographic information. This information is crucial for the blood bank not only in delivering the test results but also in reaching out to them when there is a shortage of blood inventory.
